# Parents’ Received and Expected Information About Their Child’s Radiation Exposure During Radiographic Examinations in Al Ahsa, Saudi Arabia

**DOI:** 10.7759/cureus.94810

**Published:** 2025-10-17

**Authors:** Salman S Albakheet, Batla S Al Battat, Asma s Al khofi, Fatimah S Alkhars, Rahaf T Almutairi, Noora R Bohlaiqah, Rawand A Alghannam

**Affiliations:** 1 Radiology, King Faisal General Hospital, Al-Ahsa Health Cluster, Al-Ahsa, SAU; 2 Internal Medicine, King Fahad Hospital, Al-Ahsa Health Cluster, Al-Ahsa, SAU; 3 Family Medicine, Al-Ahsa Health Cluster, Al-Ahsa, SAU; 4 Radiology, King Fahad Hospital, Al-Ahsa Health Cluster, Al-Ahsa, SAU; 5 Dermatology, King Fahad Hospital, Al-Ahsa Health Cluster, Al-Ahsa, SAU

**Keywords:** awareness, children's, kingdom of saudi arabia (ksa), radiation, radiation protection

## Abstract

Background

There is growing concern in the medical community that patients receive little or no information regarding scheduled exams and radiation. Healthcare providers did not initiate discussions about the benefits and risks of radiation from imaging tests, and most patients and parents obtained information by means of self-directed internet searches.

Aim and objectives

This study aimed to evaluate parents' awareness of radiation hazards and their experiences with the information they received from the referrer prior to their child's radiographic examination in Al Ahsa, Saudi Arabia.

Methods

A descriptive cross-sectional study included 492 parents of children who underwent radiological procedures. A self-administered survey designed and structured by researchers that was distributed to the participants online on different social media platforms, including WhatsApp (California, USA), Twitter (California, USA), and Telegram (Dubai, UAE), included questions about sociodemographic characteristics such as age, gender, and region. Furthermore, it included questions to assess the received information about the purpose and the dose of radiation.

Results

A total of 410 respondents completed the study questionnaire. Respondents' ages ranged from 19 to 65 years, with a mean age of 27.2 ± 13.9 years. The most reported source of information was healthcare staff (36.3%), followed by referral physicians (17.3%). Half of them (50.5%) reported that they received radiation information orally, while 27.6% received radiation information both orally and in written format. More than half (53.2%) of the respondents believed that scans expose their child to radiation. Only 7.8% of parents had overall good awareness regarding radiation exposure and its health-related effects on their children, while 92.2% of parents had a poor awareness level. A total of 40.3% of the parents think that they have sufficient information about the purpose of radiation, while 26.8% think that they have sufficient information about radiation dose.

Conclusion

The awareness level of parents about radiation hazards and health-related issues among children is very poor. Health personnel should thoroughly explain both risks and benefits of radiation before diagnostic procedures, either orally or in written format. In addition, community education programs should be held to raise the level of population awareness.

## Introduction

Radiology's utilization has recently been a subject of worry due to several variables such as population dose, individual dose, budgetary and financial problems, and lastly whether the examinations or justifications are appropriate [1,2]. Ionizing radiation (IR) is a noninvasive way that may be employed by various methods, such as X-ray and computed tomography (CT), to assist the treating physician in reaching a diagnosis, guiding surgical procedures, and putting a therapy plan in place. Furthermore, its usage has become a crucial aspect of diagnostic and therapeutic processes in medical practice, despite the fact that it carries potential hazards to health by increasing the risk of cancer, particularly among children [3]. CT scans have a greater radiation dosage than other imaging modalities (e.g., X-ray, fluoroscopy), and their usage has expanded in Western nations in recent years; therefore, protection is an issue [4,5]. Radiation protection in medicine is founded on the concept of justification, which seeks to ensure that the patient's benefits outweigh any short- or long-term hazards [6]. Some studies have found an increase in unnecessary CT scans among children, and the risk of getting cancer from IR exposure is greater in children than in adults, including leukemia, thyroid malignancies, and brain tumors [4,5,7]. The reason for this might be due to the growing bodies of children, the longer life expectancy after exposure to ionizing radiation (IR), and having a higher sensitivity to radiation, emphasizing the need for special attention after exposure, whether for diagnostic or therapeutic purposes. The Food and Drug Administration (FDA) recommends using potassium iodide (KI) as a radioiodine protective agent to reduce the risk of thyroid cancer following exposure [5,7]. Literature showed that the majority of parents were unaware of radiation concerns, as per the studies in Australia [8], Serbia [5], and Saudi Arabia [3]. Radiological medical practitioners (e.g., radiologists, interventional cardiologists) and other members of the radiology team (e.g., medical physicists, radiographers/radiological technicians) share responsibilities for communicating with parents. This process of teaching parents can be facilitated by the use of printed and/or electronic informational materials, and the examination's advantages and hazards should be thoroughly discussed [9-11]. There is rising concern in the medical community that patients receive little or no information regarding scheduled exams and radiation [12]. Previous research found that healthcare personnel did not initiate discussions regarding the advantages and hazards of radiation from imaging procedures, and that the majority of patients and parents learned about them through self-directed online searches [13].

The objective of this study is to assess the received and expected information of parents about their child’s radiation exposure during radiographic examinations in Al-Ahsa, Saudi Arabia.

## Materials and methods

Study design and area

This is a descriptive cross-sectional study that was conducted to assess parental knowledge regarding radiation hazards and their experience regarding information obtained from the referrer prior to their child’s radiographic examination in Al Ahsa Health Cluster, Al Ahsa, Saudi Arabia, 2022.

Study population and sample

This study investigated parents of children who underwent radiological procedures in Al Ahsa, Saudi Arabia, and had an estimated sample size of 385, based on a 0.05% marginal error and a 95% confidence interval. To enhance reliability and reduce bias, the final sample size was increased to 410. The inclusion criteria were parents of children aged 0-18 years who had undergone plain X-rays and both plain and contrast-enhanced CT scans. Conversely, the exclusion criteria were parents of patients who refused and those whose children had undergone ultrasound or MRI examinations.

Data collection tools

A self-administered online survey, created and structured by researchers. It was non-randomly distributed to participants via various social media platforms, including WhatsApp, Twitter, and Telegram. Data were collected during October and November 2022. The survey consisted of four sections: sociodemographic characteristics (such as age, gender, and region), information received about radiation exposure and dose, respondents' awareness of radiation exposure and its health effects, and their attitude and perception of the information they received (Appendix 1, Table 6). All data was securely saved in a password-protected Google database, with access restricted to investigators. A backup file was also used to ensure data preservation.

Validation and pilot study

Before the questionnaire was distributed, it underwent validation and reliability checks. Three radiologists and a biostatistician reviewed the tool to ensure content validity. Following this, a pilot study was conducted with 20 individuals who met the inclusion criteria. The results of the pilot study showed a Cronbach's alpha of more than 0.7, which confirmed the survey instrument's clarity, accuracy, and overall reliability.

Statistical analysis

The data were collected, reviewed, and then fed to IBM Corp. Released 2015. IBM SPSS Statistics for Windows, Version 21. Armonk, NY: IBM Corp. The chi-square test was employed to assess the factors associated with parents' awareness of radiation exposure and its health effects on children. All statistical methods used were two-tailed with an alpha level of 0.05, considering significance if the P value is less than or equal to 0.05. Regarding parents' awareness, each correct answer was given a 1-point score. Overall knowledge level regarding radiation dose and exposure was assessed by summing up discrete scores for different correct knowledge items. If the total score was 60% or more of the total possible score, the level of awareness was considered to be good, and scores less than 50% were considered poor. Descriptive analysis was done by prescribing frequency distribution and percentage for study variables, including parents' and children's data, source of information, and received information.

## Results

A total of 410 respondents completed the study questionnaire. Respondents' ages ranged from 19 to 65 years, with a mean age of 27.2 ± 13.9 years. Exactly 236 (57.6%) respondents were employees, while 94 (22.9%) were not working, 42 (10.2%) were students, and 38 (9.3%) were retired. As for education, 270 (65.9%) were university graduates, and 85 (20.7%) had secondary school education. As for children, the age ranged from 1 month to 18 years, with a mean age of 5.6 ± 4.8 years old. As for gender, 218 (53.2%) children were males (Table 1).

**Table 1:** Basic sociodemographic data among 410 participants N: Frequency, %: percentage

Child / parents personal data	N	%
Respondent age in years		
19-25	101	24.6%
25-40	165	40.2%
40-60	130	31.7%
61+	14	3.4%
Child age		
1-23 months	79	19.3%
2-5 years	143	34.9%
6-12 years	119	29.0%
13-18 years	69	16.8%
Child gender		
Male	218	53.2%
Female	192	46.8%
Respondent work		
Not working	94	22.9%
Student	42	10.2%
Employed	236	57.6%
Retired	38	9.3%
Educational level		
Below secondary	30	7.3%
Secondary	85	20.7%
University	270	65.9%
Post-graduate	25	6.1%

As for the received information regarding radiation exposure and dose in Al Ahsa, Saudi Arabia (Table 2), the most reported source of information was health care staff (36.3%), followed by referring physicians (17.3%), the internet (13.4%), and radiologists (9%), while 7.8% reported that they did not receive information from anyone. For the method of learning, 50.5% of participants received information orally, 27.6% received information via oral and written methods, and 13.9% received information via the written method. Regarding received knowledge about radiation, 69.3% of participants learned about the indication of radiographic examination, and 29.3% were offered other diagnostic/treatment options that do not involve radiation exposure. (24.4%) learned sufficient information about the dose of radiation involved in the scan, and it was explained using photos among 16.3% of the respondents, by numbers and scientific units among 12.7%, and using comparisons among 5.6%, while 65.4% did not use any of these methods. 

**Table 2:** Received information about radiation among 410 participants N: Frequency, %: percentage

Received information	N	%
Source of information		
Health care staff	149	36.3%
Referring physician	71	17.3%
Internet	55	13.4%
Radiologists	37	9.0%
Family / friends	37	9.0%
TV / Radio	14	3.4%
Others	15	3.7%
None	32	7.8%
Method of information delivery		
Oral	207	50.5%
Written	57	13.9%
Both	113	27.6%
None	33	8.0%
Has your doctor (or nurse or physician's assistant) told you why your child needs a scan?		
Yes	284	69.3%
No	76	18.5%
I don't know	50	12.2%
Did the doctor (or nurse or physician's assistant) who told you that your child needs a scan tell you how much radiation is involved in the scan?		
Yes	100	24.4%
No	184	44.9%
I don’t remember	126	30.7%
How was the radiation dose explained to you?		
Using numbers and scientific units	52	12.7%
Using photos	67	16.3%
Using comparisons	23	5.6%
None of the above	268	65.4%
Have you been offered any other diagnostic/treatment options that do not involve radiation exposure?		
Yes	120	29.3%
No	190	46.3%
I don’t remember	100	24.4%

In the assessment of respondents' awareness regarding radiation exposure and health effects (Table 3), 58.5% of participants didn’t know how much radiation the child would get from the scan, 53.2% expected that the test would expose the child to radiation, and 48.3% expected that radiation would affect the child's health.

**Table 3:** Parents' knowledge about radiation among 410 participants N: Frequency, %: percentage

Respondents' awareness of radiation	N	%
Based on your information, does the scan expose your child to radiation?		
Yes	218	53.2%
No	109	26.6%
I don't know	83	20.2%
How much radiation do you think your child will get from the scan compared to one year of background radiation?		
Less than 1 year	84	20.5%
Equal to 1 year	45	11.0%
More than 1 year	41	10.0%
I don't know	240	58.5%
Do you think that exposure to radiation affects your child's health?		
Yes	198	48.3%
No	100	24.4%
I don't know	112	27.3%
If yes, what is the effects?		
No effect	50	19.8%
Skin change (dryness, itching, blistering)	95	37.5%
Illness (vomiting, diarrhea, loss of appetite, fatigue)	80	31.6%
Thyroid disorders	78	30.8%
Hair loss	73	28.9%
Leucopenia	59	23.3%
Chromosomal damage	53	20.9%
Renal problem	53	20.9%
Cardiac problem	43	17.0%
Sexual disorders	24	9.5%

Regarding expected radiation health impacts, 37.5% think it’ll cause skin change (dryness, itching, blistering), followed by illness (vomiting, diarrhea, loss of appetite, fatigue) (31.6%), thyroid disorders (30.8%), hair loss (30.8%), and leucopenia (28.9%), while 19.8% think that there is no bad effect.

Only 32 (7.8%) parents had an overall good awareness regarding radiation exposure and its health-related effects on their children (Table 4). For the factors associated with parents' awareness (Table 5), only parents' education was significantly associated with their awareness level regarding radiation, as 20% of parents with a postgraduate degree had good awareness levels versus 5.9% of others with a secondary level of education (p=.046).

**Table 4:** Overall parent's awareness level regarding radiation exposure and health effect on children N: Frequency, %: percentage

Overall awareness	N	%
Good	32	7.8%
Poor	378	92.2%

**Table 5:** Factors associated with parent's awareness regarding radiation exposure and health effect on children N: Frequency, %: percentage, *: Chi square test, **: significant

Factors	Awareness level				p-value*
	Poor		Good		
	N	%	N	%	
Age in years					.327
19-25	89	88.1%	12	11.9%	
25-40	154	93.3%	11	6.7%	
40-60	122	93.8%	8	6.2%	
61+	13	92.9%	1	7.1%	
Child age					.104
1-23 months	71	89.9%	8	10.1%	
2-5 years	137	95.8%	6	4.2%	
6-12 years	105	88.2%	14	11.8%	
13-18 years	65	94.2%	4	5.8%	
Child gender					.996
Male	201	92.2%	17	7.8%	
Female	177	92.2%	15	7.8%	
Respondent work					.143
Not working	89	94.7%	5	5.3%	
Student	38	90.5%	4	9.5%	
Employed	213	90.3%	23	9.7%	
Retired	38	100.0%	0	0.0%	
Educational level					.046**
Below secondary	26	86.7%	4	13.3%	
Secondary	80	94.1%	5	5.9%	
University	252	93.3%	18	6.7%	
Post-graduate	20	80.0%	5	20.0%	
Source of information					.417
Health care staff	134	89.9%	15	10.1%	
Referred physician	66	93.0%	5	7.0%	
Internet	52	94.5%	3	5.5%	
Radiologists	32	86.5%	5	13.5%	
Family / friends	36	97.3%	1	2.7%	
TV / Radio	14	100.0%	0	0.0%	
Others	15	100.0%	0	0.0%	
None	29	90.6%	3	9.4%	
Method of information delivery					.416
Oral	191	92.3%	16	7.7%	
Written	55	96.5%	2	3.5%	
Both	101	89.4%	12	10.6%	
None	31	93.9%	2	6.1%	
Has your doctor (or nurse or physician's assistant) told you why your child needs a scan?					.124
Yes	257	90.5%	27	9.5%	
No	72	94.7%	4	5.3%	
I don't know	49	98.0%	1	2.0%	

**Figure 1:**
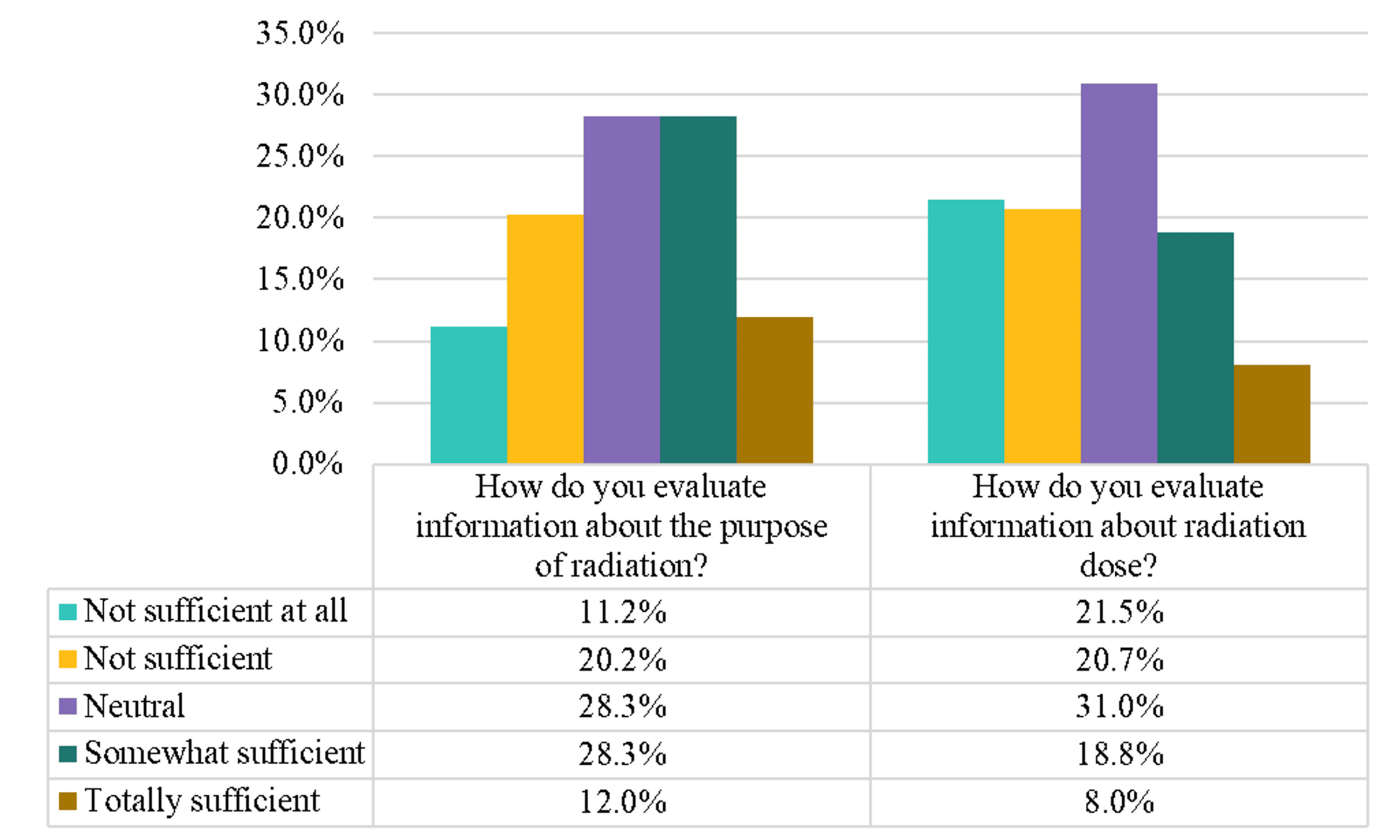
Parents' attitude and perception of received information of radiation exposure The image is created by the author.

For parents' attitude and perception of received information on radiation exposure (Figure 1). A total of 40.2% of parents think that they have sufficient information about the purpose of radiation, while 26.8% think that they have sufficient information about radiation dose.

## Discussion

Most radiological modalities use ionizing radiation, including radiography, fluoroscopy, CT, and radionuclide studies [14]. Radiation has potential biological drawbacks, such as cellular damage, and children who are radiosensitive have a higher hazard of developing certain types of cancer due to radiation exposure [9,15-17]. Hence, the term “radiation” might cause worry among the public, and events such as the Chernobyl accident further aggravate fear among them [18-21].

The current study aimed to assess the received information about the health effects of radiation exposure, the received information about the dose (amount) of radiation, and the parents’ concerns about X-ray exposure, if any, to know the source of information, and to assess the received information about the purpose of the examination. The study results showed that the most reported source of information was healthcare staff, which was reported by nearly one-third of the study parents, followed by referral physicians and the internet, while radiologists were not a common source of parents' information. Oral information was the dominant method for delivering data (nearly half of the parents), while about one-fourth received radiation information orally and in written format. Also, about two-thirds of the parents were told by their doctor (or nurse or physician's assistant) about the cause of the scan, but only one-third reported they were told about the dose of radiation, which was explained using numbers and scientific units, photos, and comparisons. Also, less than one-third of the respondents were offered other diagnostic/treatment options that do not involve radiation exposure. Similar findings were reported by Oikarinen HT et al. [22], where 83% of their study parents reported that they received satisfactory information on the purpose of the examination, 23% on other options, and 7% were told about the radiation dose. On the other hand, a study showed that radiologists explained the purpose of radiation scans, but less information was provided regarding the risks or options [9]. In a third study, the vast majority of the participants, including three parents of children with malignancy, reported that the medical staff did not initiate benefit-risk discussions [13].

As for parents' awareness of radiation exposure and associated health risks, the current study showed that parents had very poor overall awareness regarding radiation exposure and its health-related effects on their children. Nearly half of the parents think that exposure to radiation affects the child's health, such as skin change (dryness, itching, blistering), illness (vomiting, diarrhea, loss of appetite, fatigue), thyroid disorders, hair loss, and leucopenia, while 19.8% think that there is no bad effect. None of the respondents correctly reported all possible risks. A better level of parental awareness was reported by Ng CG et al. [23], where more than 40% of the respondents achieved acceptable knowledge scores by correctly answering at least six out of eleven knowledge-based questions [23]. An experimental study revealed that 66% believed CT uses radiation before reading the handout, compared to 99% afterward. Also, before reading the handout, 13% believed CT increases the lifetime risk of cancer compared to 86% afterward [24]. Hartwig HD et al. showed that 60% of caregivers did not know about the long-term negative effects of medical imaging. However, about half of the participants who did express a perceived risk from medical imaging radiation exposure could specify a known undesirable effect from exposure [12]. As compared to our study, 48.3% expected that radiation affects a child's health and were able to identify some of the potential health hazards, including thyroid disorders, leucopenia, and chromosomal damage.

Overall, only 7.8% of participants had good awareness regarding radiation exposure and its health-related effects on their children, with parents' education being the only factor that has a significant association with their awareness level regarding radiation, as 20% of parents with a postgraduate degree had good awareness levels versus 5.9% of others with a secondary level of education (p=.046).

Study limitations

This study has several limitations. Its reliance on a self-administered online survey may have introduced selection bias, targeting only parents active on social media. The cross-sectional design prevents establishing causality, and the self-reported data are subject to recall and social desirability biases. Furthermore, since the responding parent may not have been the one present during the actual clinical communication, the data may not accurately reflect the direct experience. Lastly, the findings are specific to Al Ahsa and may not be generalizable to other regions.

## Conclusions

The awareness level of parents about radiation hazards and health-related issues among children is very poor. Health personnel should thoroughly explain both risks and benefits of radiation before diagnostic procedures, either orally or in written format. In addition, community education programs should be held to raise the level of population awareness.

## Appendices

Questionnaire

**Table 6:** Questionnaire

PART 1: Child/parents personal data
Respondent age in years
19-25
25-40
40-60
61+
Child age
1-23 months
2-5 years
6-12 years
13-18 years
Child gender
Male
Female
Respondent work
Not working
Student
Employed
Retired
Educational level
Below secondary
Secondary
University
Post-graduate
PART 2: Received information
Source of information
Healthcare staff
Referring physician
Internet
Radiologists
Family/friends
TV/Radio
Others
None
Method of information delivery
Oral
Written
Both
None
Has your doctor (or nurse or physician's assistant) told you why your child needs a scan?
Yes
No
I don't know
Did the doctor (or nurse or physician's assistant) who told you that your child needs a scan tell you how much radiation is involved in the scan?
Yes
No
I don’t remember
How was the radiation dose explained to you?
Using numbers and scientific units
Using photos
Using comparisons
None of the above
Have you been offered any other diagnostic/treatment options that do not involve radiation exposure?
Yes
No
I don’t remember
PART 3: Respondents' awareness of radiation
Based on your information, does the scan expose your child to radiation?
Yes
No
I don't know
How much radiation do you think your child will get from the scan compared to one year of background radiation?
Less than 1 year
Equal to 1 year
More than 1 year
I don't know
Do you think that exposure to radiation affects your child's health?
Yes
No
I don't know
If yes, what are the effects?
No effect
Skin change (dryness, itching, blistering)
Illness (vomiting, diarrhea, loss of appetite, fatigue)
Thyroid disorders
Hair loss
Leucopenia
Chromosomal damage
Renal problem
Cardiac problem
Sexual disorders
